# Draft Genome Sequence of *Amycolatopsis mediterranei* DSM 40773, a Tangible Antibiotic Producer

**DOI:** 10.1128/genomeA.00752-14

**Published:** 2014-07-31

**Authors:** Udita Mukherjee, Anjali Saxena, Rashmi Kumari, Priya Singh, Rup Lal

**Affiliations:** Department of Zoology, University of Delhi, Delhi-110007, India

## Abstract

*Amycolatopsis mediterranei* DSM 40773 has been of special interest as successors of this strain are in use for the commercial production of rifamycin B. Here we present the draft genome sequence (~10 Mb) of this strain, which contains 108 contigs, 9,198 genes, and has a G+C content of 71.3%.

## GENOME ANNOUNCEMENT

*Amycolatopsis mediterranei* DSM 40773 was derived from the parent strain isolated from the soil of pine arboretum at St. Raphael, France (1). At the time of isolation, this strain was classified as *Streptomyces mediterranei* (1), then as *Nocardia mediterranei* (2), and finally as *Amycolatopsis mediterranei* (3). This strain has remained of inordinate interest to researchers because of its ability to produce rifamycin B, whose semisynthetic derivatives are clinically used for the treatment of tuberculosis, leprosy, and AIDS related mycobacterial infections (4). Additionally, this strain has been subjected to a classical strain improvement program and successors of this strain are in use for the commercial production of rifamycin B (5). Along with the development of cloning vectors and transformation systems (6–10), a rifamycin biosynthetic gene cluster was also characterized from this strain (11). Here we present the draft genome sequence of *A. mediterranei* DSM 40773.

The genome sequencing of strain DSM 40773 was carried out using an Illumina genome analyzer platform with the PCR-free method (12). The approximate genome size is 10,012,803 bp with over 100× genome coverage. It was assembled into 108 contigs using the ABySS 1.3.5 assembler (13) set at a k-mer size of 63. The average G+C content is 71.3%. The annotations were done on RAST server 4.0 (14) and the NCBI Prokaryotic Genomes Annotations Pipeline (PGAP) version 2 (http://www.ncbi.nlm.nih.gov/genomes/static/Pipeline.html). A total of 9,353 open reading frames (ORFs) falling into 24 clusters of orthologous groups (COGs) categories were found with COGs of the class R being most abundant. The draft genome was found to contain 9,198 genes, 9,100 coding sequences (CDSs), and 21 pseudogenes. 51 tRNA’s and 4 copies of the 16S rRNA genes were detected using RNAmmer 1.2 (15). It also contained 1,195 tandem repeats and 4 clustered regularly interspaced short palindromic repeat (CRISPR) elements.

Despite being subjected to classical strain improvement, the rifamycin polyketide synthase (PKS) gene cluster of 90 kb was identical to that of *A. mediterranei* S699 (16, 17) and *A. mediterranei* U32 (18). Analysis further revealed that other than the reported 90-kb cluster (11), DSM 40773 contains 6 other PKSs. Besides that, 12 nonribosomal peptide synthetases (NRPS)-PKS gene clusters, along with 2 hybrid NRPS/PKS gene clusters were found using NRPS Predictor 2 (19) and the PKS/NRPS Analysis website (20).

Based on average nucleotide identity, the draft genome of strain DSM 40773 was 99.99% identical to those of *A. mediterranei* S699 (16) and *A. mediterranei* U32 (18). However, the genome comparison of DSM 40773 with S699, revealed 196 single nucleotide polymorphisms (SNPs), of which there were 27 transversions and 169 transitions. It also harbors secondary metabolite gene clusters like butyrolactone, lantipeptide, terpene, bacteriocin, and ectoine.

The whole-genome sequencing of strain DSM 40773 coupled with the genome sequences of *A. mediterranei* S699, *A. mediterranei* U32, and *A. rifamycinica* DSM 46095 (21) can now be used for developing better combinatorial approaches for the production of rifamycin analogs as demonstrated recently (22). Further, a deeper insight into the genomes rifamycin B producers will aid in further understanding the mutations or changes in the genomes of the current day industrial strains that produce rifamycin B as high as ~24 g/liter (5).

### Nucleotide sequence accession numbers.

This whole-genome shotgun project has been deposited at DDBJ/EMBL/GenBank under the accession no. JMQJ00000000. The version described in this paper is the first version, JMQJ00000000.1.
